# Global meta‐analysis reveals urban‐associated behavioural differences among wild populations

**DOI:** 10.1111/1365-2656.70269

**Published:** 2026-05-18

**Authors:** Tracy T. Burkhard, Ned A. Dochtermann, Anne Charmantier

**Affiliations:** ^1^ Centre d'Ecologie Fonctionnelle et Evolutive Université de Montpellier, CNRS, EPHE, IRD Montpellier France; ^2^ Institut des Sciences de l'Evolution de Montpellier Université de Montpellier, CNRS, IRD Montpellier France; ^3^ Department of Biological Sciences North Dakota State University Fargo North Dakota USA; ^4^ Present address: Lewis & Clark College Portland Oregon USA

**Keywords:** animal personality, behavioural change, behavioural flexibility, behavioural syndrome, functional traits, phenotypic plasticity, repeatability, urbanization

## Abstract

1. Urbanization causes fundamental changes to natural environments, causing rapid and substantial adaptive phenotypic change in wild populations. While many studies have investigated how urbanization may shape *interspecific* behavioural variation, for example, via urban environmental filtering, no study has yet quantitatively assessed broad, global patterns of urban‐associated *intraspecific* behavioural variation.
2. Here, we conducted a phylogenetic meta‐analysis to assess urban‐associated behavioural differences in wild populations of birds, mammals, amphibians, reptiles and insects. We focused on four commonly measured behaviours (boldness, aggressiveness, activity, exploration) and extracted paired urban–nonurban effect size estimates for behavioural means and variances (*k* = 279), repeatability (*k* = 13) and correlations (*k* = 14) from 81 unique studies.
3. We found evidence that urban populations exhibited heightened average boldness, aggressiveness, exploration and activity compared to nonurban conspecifics, a result that was robust across geographic regions and ecological niches. However, avian species were strongly overrepresented in our meta‐analysis (*N* = 49 studies) compared to other taxa (Mammalia, *N* = 20; Reptilia, *N* = 7; Insecta, *N* = 3; Amphibia, *N* = 2). Consequently, most behaviours in non‐avian taxa were under‐sampled, and effect sizes were generally not statistically significant: among non‐avian species, only boldness differed significantly between urban and nonurban populations. This strong taxonomic bias potentially affects our other inferences as well. We did not find strong evidence linking urbanization to changes in behavioural variation, repeatability or correlations.
4. Our results summarize data from the rapidly growing field of urban evolutionary ecology and demonstrate geographically widespread differences in behaviour between urban and nonurban populations. These patterns suggest that urban populations experience parallel directional selection or that urban invaders experience environmental filtering by common urban conditions that favour certain behavioural types.
5. Taxonomic biases and methodological heterogeneity continue to limit inference in the field, constraining our ability to test preregistered hypotheses in this study. Broadening research to include understudied taxa such as invertebrates, herpetofauna and nocturnal species; incorporating common‐garden approaches; and emphasizing clearer definitions for behaviour and urbanization will yield a more comprehensive understanding of how urbanization shapes animal behaviour and other ecologically meaningful phenotypes.

## Graphical abstract

Urbanization drives rapid phenotypic change, yet broad patterns of behavioural responses remain unclear. Using a global phylogenetic meta‐analysis, we show urban populations exhibit increased boldness, aggression, exploration and activity—especially in birds—highlighting consistent behavioural shifts and revealing major taxonomic gaps that limit our understanding of behavioural adaptation to human‐altered environments

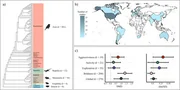

## Résumé

1. L’urbanisation entraîne des modifications fondamentales des environnements naturels, provoquant des changements phénotypiques adaptatifs rapides et substantiels au sein des populations sauvages. Bien que de nombreuses études aient examiné la manière dont l’urbanisation peut façonner la variation inter‐spécifique des comportements, notamment via le filtrage environnemental urbain, aucune étude n’a encore évalué quantitativement les tendances globales de la variation comportementale intra‐spécifique associée aux milieux urbains.
2. Dans cette étude, nous avons réalisé une méta‐analyse phylogénétique afin d’évaluer les différences comportementales associées à l’urbanisation chez des populations sauvages d’oiseaux, de mammifères, d’amphibiens, de reptiles et d’insectes. Pour quatre comportements couramment mesurés (audace, agressivité, activité, exploration), nous avons extrait des estimations appariées des tailles d’effet entre milieux urbains et non urbains concernant les moyennes et variances comportementales (*k* = 279), la répétabilité (*k* = 13) et les corrélations (*k* = 14), issues de 81 études distinctes.
3. Nos résultats indiquent que les populations urbaines présentent, en moyenne, des niveaux plus élevés d’audace, d’agressivité, d’exploration et d’activité que leurs congénères non urbains, un résultat robuste à travers différentes régions géographiques et niches écologiques. Toutefois, les espèces aviaires sont fortement surreprésentées dans notre méta‐analyse (*N* = 49 études) par rapport aux autres taxons (mammifères, *N* = 20; reptiles, *N* = 7; insectes, *N* = 3; amphibiens, *N* = 2). En conséquence, la plupart des comportements chez les taxons non aviaires sont sous‐échantillonnés et les tailles d’effet ne sont généralement pas statistiquement significatives: seule l’audace diffère significativement entre populations urbaines et non urbaines dans ce sous‐groupe d’espèces. Ce biais taxonomique marqué est susceptible d’affecter également nos autres inférences. Nous n’avons pas trouvé de preuve solide liant l’urbanisation à des changements de la variation comportementale, de leur répétabilité ou des corrélations.
4. Nos résultats synthétisent les données issues du domaine en plein essor de l’écologie évolutive urbaine et mettent en évidence des différences comportementales géographiquement répandues entre populations urbaines et non urbaines. Ils suggèrent que les populations urbaines sont soumises à une sélection directionnelle parallèle, ou que les espèces colonisant les milieux urbains subissent un filtrage environnemental lié à des conditions urbaines communes favorisant certains types comportementaux.
5. Les biais taxonomiques et l’hétérogénéité méthodologique continuent de limiter les inférences dans ce domaine, restreignant notre capacité à tester les hypothèses préenregistrées dans cette étude. L’élargissement des recherches à des taxons sous‐étudiés tels que les invertébrés, l’herpétofaune et les espèces nocturnes, l’intégration d’approches de type « jardin commun », ainsi que la mise en avant de définitions plus claires du comportement et de l’urbanisation, permettront d’obtenir une compréhension plus complète de la manière dont l’urbanisation façonne le comportement animal et d’autres phénotypes écologiquement pertinents.

## 1 INTRODUCTION

Human activity is rapidly and critically changing natural environments, leading to both unprecedented losses of species (Braje & Erlandson, 2013; Turvey & Crees, 2019; Waters et al., 2016) and alteration of local communities (McKinney, 2008; Sol et al., 2014). Within species, urbanization and its associated conditions impose survival challenges and selective pressures (Otto, 2018). These different conditions frequently correspond to behavioural changes, which are likely necessary for colonization of and survival in urban environments (Lowry et al., 2013; Miranda et al., 2013; Sol et al., 2013).

Species with certain functional traits, such as habitat or dietary generalists (Ducatez et al., 2018; Palacio, 2020; Wolf et al., 2022), thermally tolerant species (Huey et al., 2012) or carnivorous predators (Fischer et al., 2012), may be better equipped to colonize or proliferate in city environments, as predicted by the *ideal urban dweller* hypothesis (reviewed Rivkin et al., 2019). These traits have been used to classify species as urban *exploiters* and *adapters* (typically generalists) or urban *avoiders* (typically specialists), such that exploiters rely on human‐provided resources, adapters tolerate urban conditions, and avoiders do not tolerate urban resources (Blair, 1996; Bonier et al., 2007; Croci et al., 2008; McKinney, 2002; Santini et al., 2019). The *ideal urban dweller* hypothesis draws on the well‐established framework of niche theory (Carscadden et al., 2020; Chase & Leibold, 2003; Hutchinson, 1957), particularly its use of functional traits to predict species' persistence in novel or altered environments. An ‘ideal‐traits’ perspective has been widely applied in diverse fields like invasion biology (Gidoin et al., 2015; Shea & Chesson, 2002), disease ecology (Fodor & Fodor, 2011) and conservation biology (Clavel et al., 2011) to better predict which species are most likely to succeed in colonization, pathogen spread or resilience to changing environments.

More recently, niche theory has been ‘rescaled’ at both the population and individual levels, reflecting the recognition that species' niches emerge from the traits and experiences of their constituents (Carlson et al., 2021; Dall et al., 2012; Lu et al., 2025; Takola & Schielzeth, 2022). Much research has focused on behavioural traits to understand how individuals engage with their environments, thereby shaping their life experiences and defining their ecotypes. Heritable and repeatable behaviours comprising animal ‘personality’—especially boldness, exploration, aggressiveness and activity—have been particularly well studied due to their direct influence on survival probabilities (Dall et al., 2012; Lowry et al., 2013; Réale et al., 2007; Sol et al., 2013) and other fitness‐related traits, such as the affinity for dispersal, reproductive investment, willingness to sample new resources, the landscape of fear and more (Brehm & Mortelliti, 2023; Chapple et al., 2012; Cote et al., 2010; Fogarty et al., 2011; Gaynor et al., 2018; Moiron et al., 2020; Smith & Blumstein, 2008; Szabo & Ringler, 2023). In urban environments, these behaviours may be especially important as they can influence both an individual's likelihood of invading urban settings, whether passively or actively; and its ability to establish in these areas by exploiting new resources, coexisting with humans, and navigating other challenges. Accordingly, selection in urban habitats could drive behavioural divergence between urban‐dwelling populations and their nonurban counterparts (Lowry et al., 2013; Réale et al., 2007; Szulkin et al., 2020).

Reflecting this, recent empirical studies have quantified urban–nonurban differences in the four behavioural traits highlighted above. While many studies find that urban populations are bolder than nonurban conspecific populations (e.g. in birds, Ducatez et al., 2017; lizards, Baxter‐Gilbert et al., 2019; rodents, Dammhahn et al., 2020; Mazza et al., 2020), others report opposite trends or no differences (Díaz et al., 2013; Hurtado & Mabry, 2017; Mühlenhaupt et al., 2022). These inconsistencies may stem from inter‐study variation in behavioural definitions and methodologies, which complicates cross‐study comparison (Carter et al., 2013; Kaiser & Müller, 2021; Sánchez‐Tójar et al., 2022); or perhaps from true biological differences among taxa. Unfortunately, whether these patterns of urban–nonurban population differences in behaviours are generalizable remains untested.

While most studies in evolutionary ecology focus on phenotypic changes in the mean trait (Sanderson et al., 2023), urban–nonurban differences in natural selection can also result in ecologically relevant differences in phenotypic variation and its components; for example, increased among‐individual behavioural variance could allow individuals to better differentiate themselves in competitive urban environments, facilitating niche partitioning (Giraldeau, 1984; Leimar et al., 2022; Sol et al., 2021). However, whether urban and nonurban populations differ in total behavioural variation or in behavioural repeatability (i.e. the proportion of total phenotypic variance explained by among‐individual variance; Stoffel et al., 2017) has not been tested across species. Finally, whether among‐individual behavioural correlations (i.e. behavioural syndromes; Dingemanse et al., 2012), are broken, maintained, or reinforced in urban environments across species is likewise unknown (Ouyang et al., 2018). These represent significant gaps in the literature because changes in behavioural means, variations, repeatability and syndromes would provide important and complementary insights into both how behaviours are expressed in response to urbanization and their potential to evolve.

To date, no study has quantitatively compared the behaviour of urban and nonurban populations across species around the world. Limitations of previous reviews also prevented strong general conclusions. In one review, for example, Miranda and colleagues found that 6 out of 9 studies reported increased aggressiveness in urban populations and that 5 out of 6 studies reported increased boldness in urban populations (Miranda et al., 2013). Similarly, Ritzel and Gallo found that 7 out of 9 empirical studies reported increased aggressiveness, exploration and boldness in urban mammals (Ritzel & Gallo, 2020). In both reviews, however, limited sample sizes prevented statistical and phylogenetic analysis of these differences. More recently, several meta‐analyses of the literature have had greater power in detecting broad patterns and identifying emerging trends; however, though these studies have been larger in scale than previous reviews, they have had narrower scopes (e.g. focusing primarily or exclusively on single taxonomic clades [e.g. birds, (Capilla‐Lasheras et al., 2022; Samia, Møller, & Blumstein, 2015; Sepp et al., 2018); lizards, (Putman & Tippie, 2020)] or on single behaviours [Geffroy et al., 2020]); or have considered broader aspects of anthropogenic effects in addition to, or in lieu of, urbanization (e.g. captivity and domestication, [Geffroy et al., 2020]; human disturbance, [Gunn et al., 2021; Samia, Nakagawa, et al., 2015]; fishing pressure and anthropogenic noise, [Gunn et al., 2021]). As a result, we still lack generalizable conclusions about behavioural changes associated with urbanization across species on a global scale.

In this study, we conducted a phylogenetic meta‐analysis to estimate patterns of urban‐associated differences in four behavioural traits: boldness, aggressiveness, activity and exploration. To help us draw meaningful comparisons across species and study designs, we re‐interpreted behavioural data based on a common set of operational definitions (Réale et al., 2007) independent of interpretations and terminology originally used by study authors. The studies included in our meta‐analysis used observational or experimental methods to quantify behaviours, and each included at least one urban and one nonurban population. Our main goal was to focus on paired urban–nonurban comparisons of conspecific terrestrial vertebrates and invertebrates to address the following specific questions: (1) Is urbanization associated with differences in average behavioural response? (2) Is urbanization associated with differences in behavioural variation, as measured by total phenotypic variation and repeatability? (3) Is urbanization associated with changes in the direction or strength of behavioural correlations? (4) Are behavioural responses to urbanization consistent across taxa, geographic region and ecological niche?

Building on insights from previous reviews in urban ecology (Lowry et al., 2013; Miranda et al., 2013; Sol et al., 2013) within the broader context of ecological niche theory (Carscadden et al., 2020; Chase & Leibold, 2003; Hutchinson, 1957), we predicted that urban populations would exhibit heightened boldness, aggressiveness, activity and exploratory behaviour relative to their nonurban counterparts. Further, we predicted that urban populations would display greater total behavioural variation relative to nonurban conspecific populations, potentially in response to relaxed selection, increased mutation rate or developmental changes (Capilla‐Lasheras et al., 2022; Thompson et al., 2022). Likewise, we predicted that if behavioural plasticity is adaptive in heterogeneous urban habitats (e.g. Lowry et al., 2013; Sol et al., 2013), urban populations would have greater relative behavioural plasticity, as indicated by lower repeatability. We also predicted that the strength of behavioural correlations would be diminished in urban populations relative to nonurban populations. Finally, we tested whether species occupying different ecological niches had different behavioural responses to urbanization. Although theory predicts that ‘generalist’ species are more likely to inhabit urban environments, it is not yet clear whether having generalist functional traits might amplify behavioural change or instead buffer individuals from behavioural change; thus, our goal was to generate new findings.

## 2 MATERIALS AND METHODS

This study was initiated via a preregistered research plan (Open Science Framework; https://doi.org/10.17605/OSF.IO/ZF86M). This preregistration comprised our initial research questions, hypotheses, literature search results and statistical methods following literature searches to estimate base levels of data availability. Our primary deviations from our preregistration were due to being unable to perform some pre‐planned analyses given the available data after final screenings. The final data composition also required analyses examining the potential for taxonomic bias in estimation. We explain other deviations from our preregistration in the Supplemental Methods. The list of studies used in this meta‐analysis is provided in the Data Sources.

### 2.1 Systematic review and data extraction

We sought to adhere to rigorous and transparent reporting standards, modelling our methods after the PRISMA (Preferred Reporting Items for Systematic Reviews and Meta‐Analyses) guidelines. While PRISMA guidelines are widely recognized for enhancing the clarity and replicability of systematic reviews (O'Dea et al., 2021; Page et al., 2021), they are continually updated and expanded, making it challenging to establish a universally agreed‐upon standard. We aligned our methodology with PRISMA principles by emphasizing comprehensive literature searches for peer‐reviewed research, explicit inclusion and exclusion criteria, and detailed reporting of our findings (Figure S1). We provide a detailed PRISMA checklist outlining how each reporting item was addressed in our study in the Supporting Information.

As stated in the registration of predictions, searches were completed prior to preregistration to roughly determine the types and amount of data available, with secondary screening occurring after registration. We conducted four searches using the Web of Science (WoS) Core Collection on September 8, 2022, limiting each search to the Science Citation Index Expanded database (SCI‐EXPANDED, 1900—present). We selected the most popular methods and keywords in behavioural and urban ecology research as search terms for our study to provide a representative overview of the field. Full details and search terms can be found in the Appendix A. These Web of Science literature searches produced 1323 studies.

In addition to our literature searches, we identified relevant studies by checking three published reviews and one published meta‐analysis (*N* = 17 relevant papers from Lowry et al., 2013); 43 from (Ritzel & Gallo, 2020); 3 from (Thompson et al., 2022); and 20 from (Gunn et al., 2021). We made the strategic decision to focus on the bibliographies of reviews for study identification, but not from empirical papers, due to both the considerable overlap in the papers cited among reviews and those identified in our literature search and the sheer volume of (mostly redundant) papers we would have had to screen. Reviews typically synthesize large bodies of work and often highlight key papers that have shaped the field; this allowed us to quickly identify any robust empirical study missed by our WoS search within the constraints of time and resources.

Different search engines (e.g. Google Scholar, WoS) recover incomplete or inconsistent lists of articles due to institutional licensing, biases towards research from Western countries and more (Gusenbauer, 2022; Pozsgai et al., 2021; Tennant, 2020). Thus, we also included 4 relevant papers identified by a colleague (MJ Thompson) that were not identified by WoS.

We de‐duplicated our cumulative list of studies from literature search, bibliography review and word‐of‐mouth using the ‘Remove Duplicates’ function in Excel (Microsoft Office), followed by visual inspection of the author lists and titles. De‐duplication resulted in a list of 1086 unique studies (Figure S1; Table S1).

Studies were excluded if they did not meet our preregistered inclusion criteria (Supporting Information Methods; Figure S1; Table S1). We also identified papers that analysed the same or overlapping datasets, such as those from studies by the same lab or sets of authors that analysed a longitudinally growing dataset. For these datasets (*N* = 8 studies), we included only the study using the dataset with the largest sample size, which was in all cases the most recently published study. If a study met the screening criteria, we extracted behavioural trait means, standard deviations (SD) and sample sizes (N) from the text, tables or supplemental files. For cases where the standard error (SE), but not SD, was provided, we calculated standard deviation as SD = SE*$\sqrt{N}$, where *N* was the sample size. If these statistics were not available within either the article or supplemental files, we contacted the corresponding author(s) of the study for these estimates (*N* = 10). Authors of eight of these ten studies provided us with raw data or the necessary statistics and were subsequently included in analyses. When authors did not respond to our request (*N* = 2), we extracted mean and standard deviation or error estimates from available figures using a digital ruler (Van der Mierden et al., 2021). When available, we also extracted available repeatability coefficients (*τ*, unadjusted or adjusted for fixed effects, with preference for unadjusted repeatabilities, Nakagawa & Schielzeth, 2010) and phenotypic correlation coefficients (*r*).

Screening and estimate extraction were conducted by the first author following a standardized protocol to ensure consistency and minimize variability in data extraction. In 2025, the first and last authors independently re‐screened a subset of 50 papers to determine the potential for inclusion bias. This rescreening demonstrated low potential for such bias: there were only two discrepancies between the initial and repeat screenings. One discrepancy involved a study that was included in the subset screening but excluded in the full screening due to its dataset contributing to a larger, more recent dataset by the same authors. The second discrepancy was resolved through discussion after joint review of the full text and the screening criteria.

In addition to extracting quantitative behavioural data, we recorded the following information for each study: common species name, species name, taxonomic class, geographic region, behavioural assay (e.g. open field test, flight initiation distance), behavioural metrics (e.g. distance run, quadrants explored, latency to flee), number of urban and nonurban populations, and whether behaviours were measured from wild animals tested or observed in the field, wild‐caught animals tested in captivity, or from wild‐derived animals bred and tested in a common environment.

#### 2.1.1 Evaluation of search robustness and potential database biases

To evaluate whether our original Web of Science search may have systematically excluded relevant studies, we conducted two rounds of supplemental literature searches following peer review.

Following an early round of peer review, we conducted a supplemental Web of Science search on 28 January 2025 to address omissions in the original search—specifically, the absence of terms related to behavioural repeatability (e.g. ‘repeatability’, ‘among‐individual variation’, ‘individual consistency’). This updated search yielded 11 additional records. Two were newly published after our original search window and thus were not eligible for inclusion. Seven were duplicates of previously identified studies. The two novel records focused solely on within‐urban repeatability estimates without urban–nonurban comparisons and were therefore not eligible under our inclusion criteria. This follow‐up confirms that our original dataset is unlikely to have systematically excluded studies meeting our inclusion criteria, including those involving repeatability, despite the inadvertent initial omission of certain search terms.

Following the penultimate round of peer review, we conducted three supplemental literature searches in SCOPUS on 28 February 2026 to assess whether our restriction to Web of Science might have systematically excluded relevant studies. Searches were designed to replicate our original strategy using Web of Science as closely as possible, while accounting for differences in syntax, subject area categories, and search operators. We focused on the main search—studies assessing animal behaviour in relation to urbanization and human disturbance—and on keywords related to behavioural repeatability, as suggested in an earlier round of peer review. Because none of the authors' home institutions have subscriptions to SCOPUS, a limitation that informed our preregistered study design, we borrowed access through a neighbouring institution (Reed College, Portland, Oregon) to conduct the supplemental search. This underscores a broader challenge for meta‐analysts at smaller institutions or in resource‐limited settings, where access to multiple databases may not be feasible. Our SCOPUS searches returned 213 results, of which 89 were duplicates, leaving 124 unique papers. One study was excluded because its publication date (December 2022) was after our original search (September 2022), thus leaving 123 papers. Of these, 59 had already been captured in our WoS searches, an overlap of 48%. Screening the remaining 64 studies according to our inclusion criteria identified only one additional eligible study. These results suggest that differences in database coverage are minimal and would not meaningfully alter the patterns observed in our meta‐analysis. Additional details can be found in the Supporting Information Methods.

### 2.2 Defining behavioural categories

We initially focused on the behavioural traits of boldness, aggression, activity, exploration and sociability because of the rich literature establishing their role on survival and reproductive success in a wide range of species (Koolhaas et al., 1999; Réale et al., 2010; Smith & Blumstein, 2008). Our literature searches returned no articles with observations of sociality in an urban context, so we omitted sociality from our analyses. We adopted definitions for the remaining four behaviours following (Réale et al., 2007): we defined ‘boldness’ as individual reactions to risk, ‘exploration’ as reactions to novel situations in the absence of risk, ‘activity’ as the amount of movement of an individual and ‘aggressiveness’ as agonistic reactions by individuals to conspecifics. Note that under our study's operational definitions, aggressive responses towards predators or humans are interpreted as measures of boldness.

### 2.3 Quantifying urbanization

Urbanization can be assessed by a multitude of factors, including human population density, infrastructure development, resource availability, pollution levels and land use patterns (Melliger et al., 2018; Moll et al., 2019, 2020). In our literature review, we were unable to find a metric for urbanization that was used consistently across studies. For example, out of the 80 studies comprising our final standardized mean differences (SMD) dataset, 20 studies defined urbanization as percent impervious cover, 8 defined urbanization as human population per km^2^, and 3 defined urban and nonurban populations as relative differences in total human population density. Although we initially aimed to examine urban‐associated phenotypes along explicit urbanization gradients (lines 256‐257 of preregistration), only 23 studies included both multiple urban and nonurban populations and a quantitative measure of urbanization (e.g. impervious surface cover, human population density). Thus, rather than limiting our dataset to only those studies using a certain definition for urbanization, we instead relied on designations of ‘urban’ and ‘nonurban’ environments provided by the authors.

### 2.4 Collection of moderator data

We classified species as urban exploiters, adapters or avoiders based on published designations in birds (Palacio, 2020, which expands on data from Bonier et al., 2007; Croci et al., 2008) and mammals (Santini et al., 2019). While the avian dataset used only two categories, adapters and avoiders, we reclassified highly urban‐tolerant species (e.g. *Passer domesticus*, *Columba livia*) as urban exploiters, thus allowing consistency with the mammalian dataset, which used all three categories. These decisions were made by consulting studies that explicitly applied this category to certain species (e.g. Johnson & Munshi‐South 2017; Sol et al., 2020). In practice, these categories are often assigned by presence/absence data (Rodriguez et al., 2021; Santini et al., 2019) and come with caveats, particularly regarding the challenges of defining ‘urban’ environments and mislabelling species (Fischer et al., 2015). Further, we note that by our own criteria, all species included in our study were present in both urban and nonurban habitats. Nonetheless, we find these categories useful as they provide a simple framework for understanding how behavioural responses may differ among taxa with different tolerances of urbanization.

To categorize each species' ecological niche, we collected data on dietary type (e.g. omnivore, carnivore, plant and seed, fruit and nectar or invertebrate), dietary category (specialist or generalist) and temporal activity type (nocturnal, diurnal or cathemeral) from open‐access databases, using the EltonTraits 1. 0 database (Wilman et al., 2014) for birds and mammals, the ‘amphibio’ dataset from the traitdata R package for amphibians and reptiles (version 0.0.1, Meiri, 2018, 2019; Oliveira et al., 2017a, 2017b; RS‐eco, 2022), and published datasets for invertebrates (Digweed, 1994; Luff, 1978; Stone et al., 2002; Tseng et al., 2018; Vucic‐Pestic et al., 2010; Williams, 1959; Zygmunt et al., 2006).

### 2.5 Calculation of effect sizes

We calculated between‐group standardized mean differences (SMD, Hedges, 1981) and differences in log standard deviation (∆ln(SD), Nakagawa et al., 2015) to investigate differences in mean behavioural traits and total behavioural variation, respectively. We calculated SMD as Hedges' *g* corrected for bias and sample size (Hedges, 1981) using the metafor package (v 4.8.0, Viechtbauer, 2010). We calculated differences in ln(SD) as the unbiased estimator of the natural logarithm of the population standard deviation (ln σ) following (Nakagawa et al., 2015):

(1) $$\ln\;\hat{\sigma }=\ln s+\frac{1}{2\left(n-1\right)},$$

(2) $$s_{\ln\;\hat{\sigma }}^{2}=\frac{1}{2\left(n-1\right)}.$$

In total, we were able to calculate 286 effect sizes for both SMD and ∆ln(SD). Of these, we excluded 7 effect sizes because they had standard deviations equal to 0, ending with a final set of 279 effect sizes for both SMD and ∆ln(SD) (Figure S1). To allow testing of our directional predictions, we adjusted the sign of effect size estimates such that positive values indicated higher mean values in urban populations (e.g. heightened boldness, exploration, activity or aggressiveness). In total, we changed the sign of 211 of 279 effect sizes (75.6%).

When available, we also recorded paired urban–nonurban estimates of repeatability (*τ*) and behavioural correlations (*r*). Due to a small sample size of reported repeatability coefficients, we pooled unadjusted and adjusted repeatability coefficients in our analysis of repeatability. Likewise, we pooled between‐individual correlation coefficients and total phenotypic correlations for analyses of behavioural correlations. Repeatability and correlation coefficients were transformed for analyses using Fisher's *z* transformation (Fisher, 1921) and back‐transformed for interpretation.

### 2.6 Data analysis

Meta‐analyses of standardized mean differences (SMD), repeatability and correlation coefficients were run in R using the metafor package, with visualizations produced using metafor, ggplot2 (v 3.5.1, Wickham, 2016) and the orchaRd package (v 2.0, Nakagawa et al., 2021). Each model was fitted assuming unstructured variance–covariance matrices. For analyses comparing ∆ln(SD), we used the MCMCglmm package (v 2.34, Hadfield, 2010). Full details for MCMCglmm models can be found in the Supplemental Materials. For all models, we considered estimates to be statistically supported if they had a 95% confidence interval or, for Bayesian models, 95% highest density posterior intervals (HDPI), that did not overlap zero. Phylogenetic trees to account for non‐independence were built using phyloT (Letunic, 2015) and the rotl package (v 3.1.0, Michonneau et al., 2016).

#### 2.6.1 Intercept‐only models

We fitted phylogenetic meta‐regression models with an intercept but no moderators to estimate the global effect size of urbanization for each component of behavioural differences (i.e. SMD, ∆ln(SD), repeatability and behavioural correlations). We first fitted models that pooled behaviour type for analyses of mean, behavioural variability, repeatability and behavioural correlation combinations (e.g. aggressiveness‐boldness, exploration‐activity) for analyses of behavioural syndromes, thus estimating a global effect size for a general behavioural response, not for each behavioural type. We then fitted separate intercept‐only models by behavioural type for analyses of mean, variance and repeatability. For each intercept‐only model, we included four random effects: study identity, observation identity, species and phylogeny. Due to the limited and imbalanced sample sizes for each behavioural combination, we refrained from fitting behavioural‐combination‐specific models for syndromes.

**FIGURE 1:**
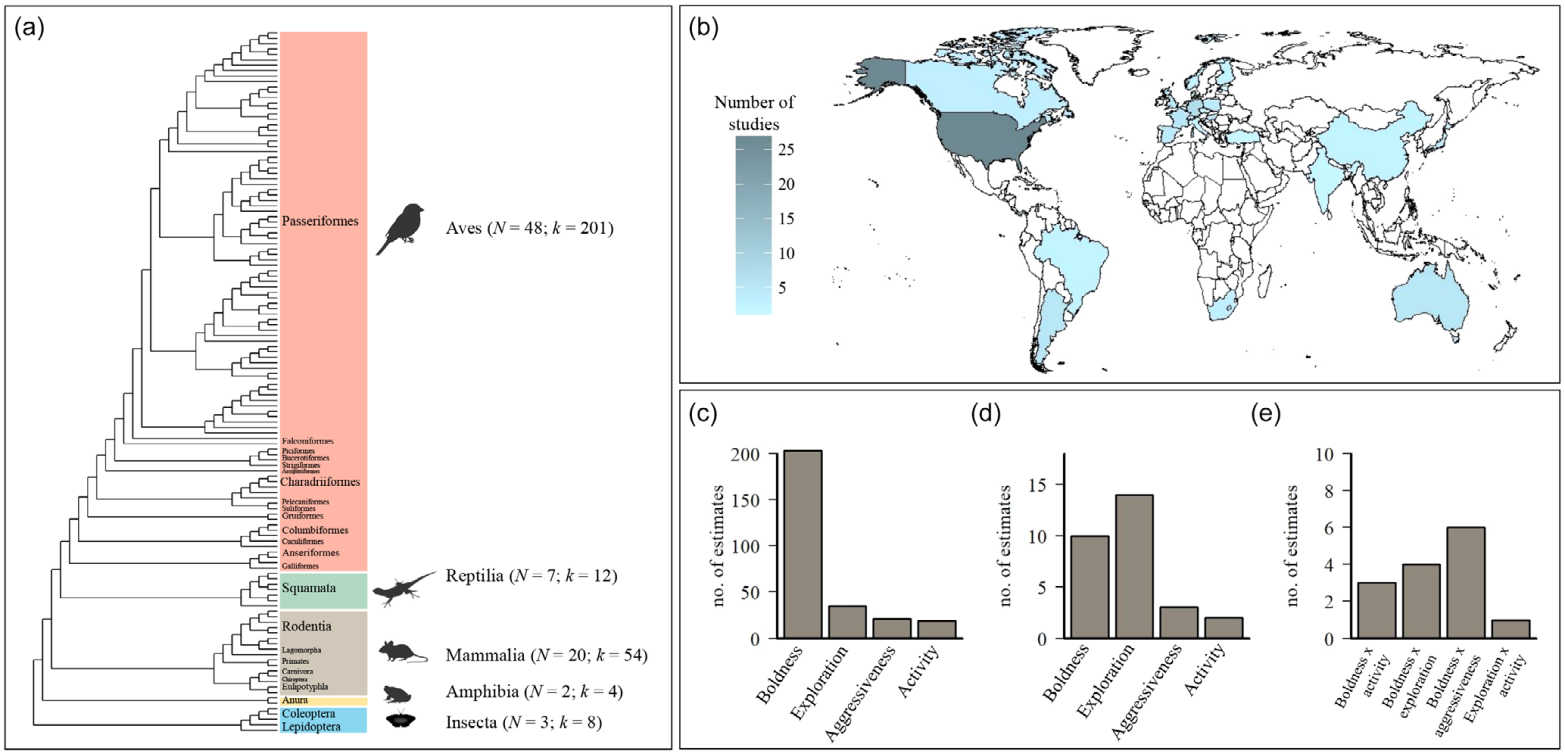
Taxonomic, behavioural and geographic breadth of the meta‐analysis. (a) Phylogenetic tree depicting all species represented in the 80 papers included in the meta‐analysis of standardized mean differences (SMD). Branches are annotated with taxonomic orders represented in the study and colour‐coded by taxonomic class. Labels indicate sample sizes for unique studies (*N*) and effect size estimates (*k*) by taxonomic class. The full list of species can be found in the Supplemental Information. (b) World heatmap depicting the number of studies conducted in each country. Geographic coverage was uneven, with many countries lacking data and a strong bias towards North American and European populations. (c–e) Bar charts showing the number of paired urban–nonurban observations for each behavioural category included in the meta‐analysis: (c) behaviours included in SMD and log standard deviation difference analyses; (d) behaviours included in repeatability analysis; and (e) behavioural combinations included in syndrome analysis.

Finally, to better understand how birds, the primary taxa in our data set (Figure 1a), affected our results, we conducted a complementary sub‐group analysis wherein we fitted separate intercept‐only models for SMD and ∆ln(SD) datasets for: (1) Aves only and (2) non‐avian taxa.

#### 2.6.2 Moderator meta‐regression models

For the SMD and ∆ln(SD) datasets, which had the largest sample sizes, we tested whether the relationship between urbanization and behavioural response differed among a priori selected moderators. We first fitted a ‘full’ model that included each of the following moderators: region, provenance of data (whether the study populations were wild animals observed in the field, wild animals observed in captivity, or wild‐derived animals bred and observed in captivity), urban tolerance, diet type, diet specialism and temporal activity (Figure S1). The full model was fitted on the largest available subset of observations with complete information for each of the moderators, comprising 163 effect sizes from 40 studies for SMD and for ∆ln(SD). As some moderator categories had few estimates, or categories had uneven sample sizes (e.g. if the dataset was composed entirely of observations of birds and mammals), we also tested each moderator in separate meta‐regressions (Royauté et al., 2018; Vincze et al., 2017). All models had the same random effects as the global intercept‐only meta‐analyses.

#### 2.6.3 Heterogeneity

Heterogeneity estimates the consistency of the effect sizes, which can be inflated if studies greatly differ methodologically, taxonomically or in geographic region (Higgins & Thompson, 2002; Higgins et al., 2003). For analyses done in metafor, we estimated heterogeneity (*I* ^2^) using the *i2_ml* function in the orchaRd package (v 2.0, Nakagawa et al., 2021), following (Nakagawa & Santos, 2012; Senior et al., 2016). To calculate heterogeneity (*I* ^2^) of Bayesian models, we divided the modal estimated variance by the total variance in the data (Nakagawa & Santos, 2012). We considered *I* ^2^ values around 25%, 50%, and 75% as low, moderate and high heterogeneity respectively, following convention set by (Higgins et al., 2003).

#### 2.6.4 Publication bias

Publication biases can occur when large effect sizes are overrepresented in the literature, that is, small‐study bias, or when effect sizes diminish over time as published studies begin showing reduced support for a given hypothesis, that is, time‐lag bias (Jennions & Møller, 2002; Nakagawa et al., 2022). We visually inspected funnel plots and ran mixed‐effects meta‐regression model tests to assess asymmetry (Sterne & Harbord, 2004). Because many traditional methods for detecting publication bias (e.g. funnel plots, Egger's regression, time‐lag regression) struggle with ecological datasets exhibiting high heterogeneity and non‐independence (Nakagawa et al., 2022) or may be inappropriate for multilevel meta‐analysis models (e.g., trim‐and‐fill methods), we followed Nakagawa et al.'s (2022) recommendations for fitting multilevel meta‐regression models (Nakagawa et al., 2022). We included two moderators: the square‐root of the sampling variance, to test for small‐study bias; and the mean‐centred year of study publication, to test for time‐lag bias (Nakagawa et al., 2022). We did not test or qualitatively assess included studies for other forms of bias.

## 3 RESULTS

### 3.1 Summary of datasets

Our final datasets included effect sizes from 81 unique studies (Figure S1; Data Sources). Of these studies, 80 studies reported 279 effect sizes for standardized mean differences (SMD) and for log standard deviation differences (∆ln(SD)); 13 studies reported 29 paired urban–nonurban repeatability coefficients (*τ*), and 14 studies reported 14 paired urban–nonurban correlation coefficients (*r*). The studies reporting repeatability and syndrome coefficients were a subset of the 80 studies we analysed for SMD and ∆ln(SD) with the exception of one study (Charmantier et al., 2017) included exclusively in the repeatability analysis.

Though the 80 studies included in SMD and ∆ln(SD) analyses spanned 133 different species, birds comprised 72.0% of all observations (*k* = 201, Figure 1a). By contrast, mammals comprised 19.4% of total observations (*k* = 54), while collectively, ectothermic species comprised fewer than 10% of total observations (amphibians, 1.4% (*k* = 4); insects, 2.9% (*k* = 8); reptiles, 4.3% (*k* = 12)). Our dataset was also strongly biased towards studies conducted in Europe (62.7%) and North America (18.3%) (Figure 1b). Although we initially intended to partition data into class‐specific datasets and run sub‐group meta‐analyses separately for each clade (lines 266–267 of preregistration), uneven taxonomic representation precluded separate analyses by class. Instead, effect sizes were partitioned into two groups to ensure sufficient sample sizes for comparison: an avian‐only dataset and a non‐avian dataset comprising mammals, invertebrates, amphibians and reptiles.

Behavioural trait types were also unevenly represented across studies (Figure 1c–e). For SMD and ∆ln(SD), boldness was the best studied behavioural trait (*k* = 204 effect sizes, 73.1% of total effect sizes), followed by exploration (35, 12.5%), activity (21, 7.5%), and aggressiveness (19, 6.8%). This bias for boldness was directly caused by the taxonomic bias favouring birds, as 170 of the boldness effect sizes (83.3%) were made from observations in birds; of these, 161 (94.7%) were from measures of flight initiation distance (FID), which is a relatively easy and common field measure of boldness in birds. Thus, 78.9% of all boldness estimates consisted of FID measured in birds. Excluding avian taxa, behavioural traits were more equally represented in our SMD and Δln(SD) datasets: out of 78 total effect sizes, 34 were for boldness (43.6%), 21 were for exploration (26.9%), 20 were for activity (25.6%), and 3 were for aggressiveness (3.8%). Most of the observations in our study came from wild animals measured in the field (*k* = 207) or wild‐caught animals measured in captivity (*k* = 64), with few observations coming from wild‐derived animals reared and tested in a common garden (*k* = 8).

Among the 13 studies that reported paired urban–nonurban repeatability estimates, exploration and boldness were the best documented (exploration: 14 paired estimates, 48.3% of paired estimates; boldness: 10, 34.5%), followed by activity (3, 10.3%) and aggressiveness (2, 6.9%). Of these 29 paired repeatability estimates, 17 were reported as adjusted repeatability, 8 were reported as unadjusted, and 4 were unspecified.

Ten studies reported correlation coefficients for different combinations of behavioural traits. Of these, 8 studies reported behavioural correlation coefficients for both nonurban and urban populations. In total, we recovered 14 paired urban–nonurban correlation coefficients from the 8 studies, 3 of which were reported as between‐individual correlation coefficients, and 11 of which were reported as total phenotypic correlations. Of the behavioural combinations reported, we recovered 6 paired correlation coefficients for aggressiveness and boldness, 4 for boldness and exploration, 3 for activity and boldness, and 1 for activity and exploration.

### 3.2 Is urbanization associated with changes in average behaviours?

**FIGURE 2:**
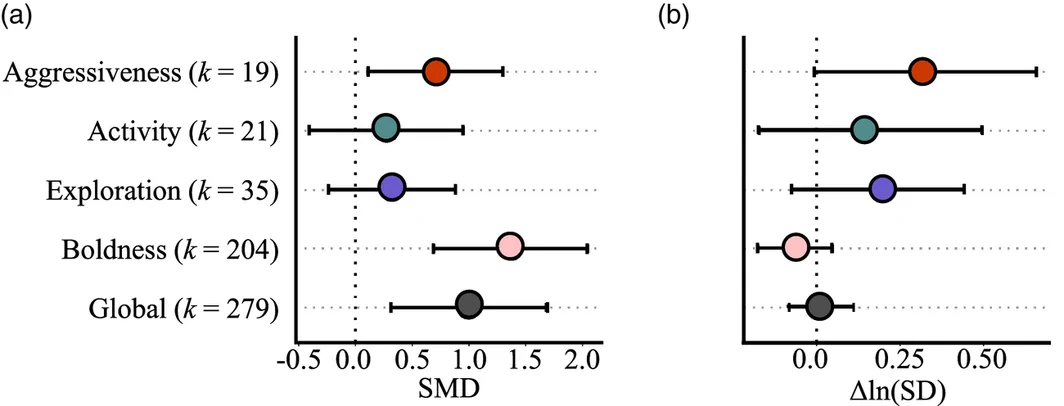
Forest plots of estimates for (a) standardized mean differences, SMD, and (b) differences in log standard deviation, ∆ln(SD). (a) Urban populations have greater mean behavioural responses than nonurban populations across behaviours, but especially boldness and aggressiveness. (b) Conversely, urban populations do not differ from nonurban populations in behavioural variation. Positive values on the *x*‐axis indicate higher mean (a) or variation (b) in urban populations than in nonurban populations. Black error bars indicate (a) 95% confidence intervals (for SMD) or (b) 95% credible intervals calculated from highest posterior density interval (HDPI, for ∆ln(SD)). In both panels, *k* provides the number of paired urban–nonurban estimates. Orchard plots with the meta‐analytic estimates and raw data in Figures S2 and S3.

Global meta‐analysis revealed a positive standardized mean difference (SMD) effect size, indicating that urban populations expressed greater behaviours (i.e. more aggressiveness, more activity) on average than nonurban populations (SMD estimate [95% CI] = 1.00 [0.31, 1.68]; Figure 2a; Table 1). Urban populations were both more aggressive (SMD [95% confidence interval] = 0.70 [0.11, 1.30]) and bolder (1.37 [0.69, 2.05]) than nonurban populations. Urban populations also tended to be more active (0.27 [−0.41, 0.95]) and more explorative (0.32 [−0.24, 0.88]) than nonurban conspecifics, though confidence intervals for both overlapped zero.

**TABLE 1:** Estimates of SMD and ∆ln(SD) from meta‐analytic models for: (1) all taxa, (2) avian taxa, (3) non‐avian taxa.

Species	Moderator	*k*	SMD [95% CI]	∆ln(SD) [95% HDPI]
(1) All taxa	Global	279	*1.00* [*0.32, 1.68*]	0.01 [−0.08, 0.11]
	Boldness	204	*1.37* [*0.70, 2.04*]	−0.06 [−0.17, 0.05]
	Activity	21	0.27 [−0.41, 0.95]	0.14 [−0.17, 0.50]
	Aggressiveness	19	*0.70* [*0.11, 1.30*]	0.32 [−0.01, 0.66]
	Exploration	35	0.32 [−0.23, 0.87]	0.20 [−0.07, 0.44]
(2) Avian species	Global	201	*1.59* [*0.93, 2.25*]	−0.08 [−0.22, −0.08]
	Boldness	170	*1.78* [*1.03, 2.54*]	−0.14 [−0.32, 0.01]
	Activity	1	*1.54* [*0.11, 2.96*]	−0.02 [−1.95, 2.00]
	Aggressiveness	16	*1.32* [*0.71, 1.93*]	0.44 [−0.07, 0.94]
	Exploration	14	*0.94* [*0.35, 1.53*]	0.38 [−0.17, 0.94]
(3) Non‐avian species	Global	78	0.51 [−0.04, 1.05]	0.07 [−0.24, 0.39]
	Boldness	34	*1.09* [*0.44, 1.75*]	0.02 [−0.38, 0.46]
	Activity	20	−0.26 [−0.63, 0.11]	0.17 [−0.36, 0.73]
	Aggressiveness	3	0.07 [−0.14, 0.28]	−0.31 [−1.81, 1.1]
	Exploration	21	−0.11 [−0.42, 0.21]	0.08 [−0.46, 0.65]

*Note*: Effect size estimates are shown with associated 95% confidence intervals or 95% highest density posterior intervals (HDPI). *k* indicates the number of observations for each moderator. Statistically supported estimates with 95% intervals not overlapping zero are in italics.

Results for the avian subset of data likewise showed that urban populations of birds exhibited significantly greater averages across all four behaviours compared to nonurban populations (though note the estimate for activity was derived from a single study; Tables 1 and 2). Conversely, excluding birds from the analysis dampened the relationship between behavioural response and urbanization, though non‐avian urban populations still tended to express greater average behaviours than nonurban populations (SMD [95% CI] = 0.51 [−0.04, 1.05]; Tables 1 and 2). This result was driven by boldness, which was the only behaviour that significantly differed between urban and nonurban non‐avian populations (1.09 [0.44, 1.75]).

**TABLE 2:** Estimates of SMD and ∆ln(SD) from moderator meta‐regression models.

Category	Moderator	*k*	SMD (95% CI)	∆ln(SD) [95% HDPI]
Taxonomic class	Amphibia	4	−0.07 [−2.55, 2.42]	−0.11 [−0.91, 0.61]
	Aves	201	*1.59* [*0.69, 2.49*]	−0.01 [−0.12, 0.11]
	Insecta	8	0.18 [−1.96, 2.32]	−0.08 [−0.58, 0.40]
	Mammalia	54	0.72 [−0.67, 2.11]	0.04 [−0.19, 0.23]
	Reptilia	12	0.13 [−1.56, 1.82]	0.28 [−0.17, 0.70]
Diet type	FruiNect	12	0.77 [−0.13, 1.68]	0.03 [−0.39, 0.45]
	Invertebrate	66	*0.94* [*0.21, 1.68*]	−0.15 [−0.32, 0.05]
	Omnivore	103	*1.15* [*0.43, 1.86*]	0.06 [−0.10, 0.22]
	PlantSeed	71	*0.9* [*0.15, 1.65*]	0.14 [−0.03, 0.33]
	VertFishScav	27	*0.87* [*0.08, 1.66*]	−0.15 [−0.46, 0.15]
Diet type	Generalist	169	*1.1* [*0.4, 1.8*]	0.05 [−0.09, 0.20]
	Specialist	110	*0.91* [*0.22, 1.61*]	−0.02 [−0.13, 0.11]
Population	Common garden	8	0.38 [−1.23, 1.99]	−0.1 [−0.61, 0.42]
	Field	207	*1.38* [*0.68, 2.09*]	−0.07 [−0.18, 0.04]
	Wild‐caught	64	0.5 [−0.26, 1.27]	*0.24* [*0.06, 0.43*]
Diel activity	Cathemeral	17	0.6 [−0.31, 1.51]	0.1 [−0.30, 0.48]
	Diurnal	244	*1.09* [*0.4, 1.78*]	0.01 [−0.10, 0.11]
	Nocturnal	18	0.75 [−0.22, 1.72]	−0.07 [−0.45, 0.29]
Thermoregulation	Ectotherm	24	0.07 [−1.07, 1.22]	0.1 [−0.20, 0.41]
	Endotherm	255	*1.34* [*0.6, 2.09*]	0 [−0.09, 0.10]
Urban tolerance	Adapter	88	*0.84* [*0.06, 1.63*]	0.01 [−0.15, 0.16]
	Avoider	17	0.5 [−0.35, 1.35]	*−0.48* [*−0.89, −0.10*]
	Exploiter	58	*0.93* [*0.22, 1.64*]	0.12 [−0.07, 0.33]

*Note*: Effect size estimates are shown with associated 95% confidence intervals (SMD) or 95% highest density posterior intervals (∆ln(SD)). For all categories except Urban tolerance, the total number of paired urban–nonurban observations is 279; for Urban tolerance, the total number is 163. Statistically supported estimates with 95% intervals not overlapping zero are in italics.

### 3.3 Is urbanization associated with changes to behavioural variation?

Urban populations did not differ significantly in behavioural variation from nonurban populations (∆ln(SD) [95% credible intervals]: 0.01 [−0.08, 0.11]; Figure 2b; Table 1). Urban populations tended to have more behavioural variation than nonurban populations in aggressiveness (0.32 [−0.01, 0.66]), activity (0.14 [−0.17, 0.50]) and exploration (0.20 [−0.07, 0.44]), though they exhibited slightly less variation in boldness relative to nonurban populations (−0.06 [−0.17, 0.05]). Importantly, however, Bayesian credible intervals for all behaviours overlapped zero, suggesting that these differences were not strong. We repeated these analyses in avian‐only and non‐avian‐only subsets; differences in magnitude of phenotypic variation were not strong in either subset (Tables 1 and 2).

**FIGURE 3:**
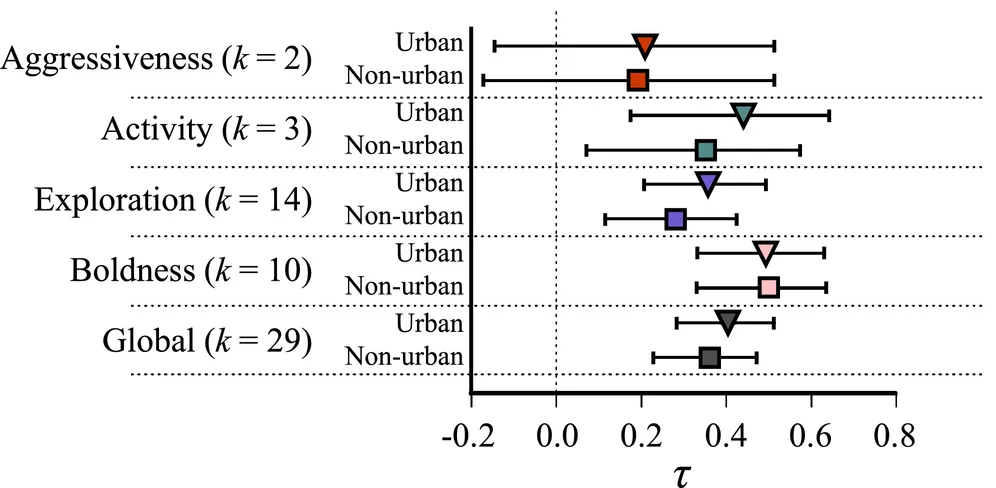
Forest plot of estimates for repeatability correlation coefficients, *τ*, for urban (inverted triangles) and nonurban (squares) populations. Black error bars indicate 95% confidence intervals; *k* provides the number of paired urban–nonurban observations.

We next focused on pairwise urban–nonurban comparisons of repeatability (Figure 3, Table S2). Both urban and nonurban populations had moderately strong repeatability across behaviours, with urban populations having slightly greater repeatability than nonurban populations (back‐transformed *τ* [95% confidence intervals]: urban *τ*: 0.41 [0.29, 0.51]; nonurban *τ*: 0.36 [0.24, 0.47]). The confidence intervals for both estimates overlapped substantially, suggesting that any true difference is likely small or not biologically meaningful. Models parsing out specific behaviours corroborated this result, revealing that while repeatability estimates for individual behaviours were similar in strength between urban and nonurban populations, with urban populations tending to have greater repeatability, confidence intervals for each estimate overlapped substantially. Repeatability differed among behaviours, with boldness having the highest repeatability for both urban and nonurban populations (back‐transformed *τ* [95% CI]; urban *τ*: 0.49 [0.33, 0.63]; nonurban *τ*: 0.50 [0.33, 0.64]). Repeatability was moderately high for activity (urban *τ*: 0.44 [0.18, 0.64]; nonurban *τ*: 0.35 [0.07, 0.57]) and exploration (urban *τ*: 0.35 [0.20, 0.49]; nonurban *τ*: 0.28 [0.12, 0.42]). Aggressiveness, which had the fewest paired urban–nonurban estimates (*k* = 2), had moderate repeatability and confidence intervals that overlapped zero (urban *τ*: 0.21 [−0.14, 0.51]; nonurban *τ*: 0.19 [−0.17, 0.51]).

### 3.4 Do nonurban and urban populations differ in behavioural correlations?

**FIGURE 4:**
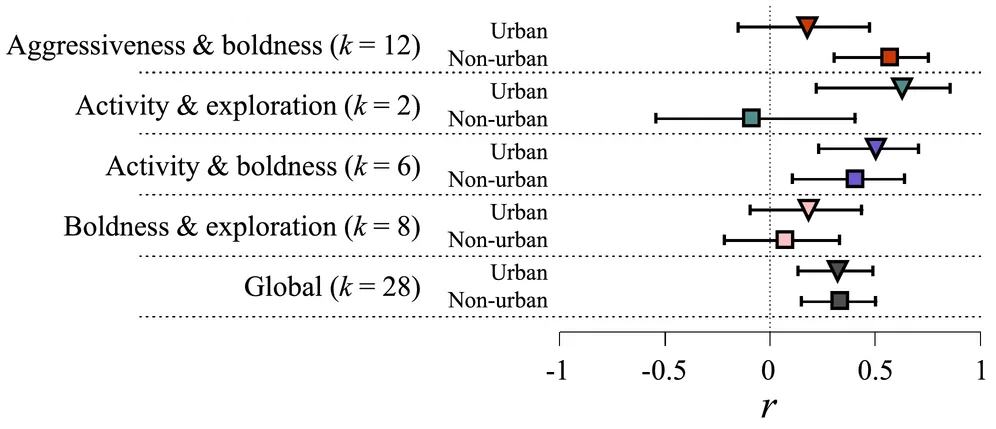
Forest plot of estimates for behavioural correlations, *r*, for urban (inverted triangles) and nonurban (squares) populations. Black error bars indicate 95% confidence intervals; *k* provides the number of paired urban–nonurban observations.

On average, behaviours were moderately and positively correlated in both urban and nonurban populations, and the magnitude of behavioural correlations did not differ substantially between the two habitat types (back‐transformed *r* [95% CI]; urban, 0.32 [0.13, 0.49]; nonurban, 0.34 [0.15, 0.50]; Figure 4; Table S3).

Across the 14 urban–nonurban paired estimates, four differed in sign between urban and nonurban populations, with two having negative coefficients in urban populations but positive coefficients in nonurban populations, and with two having positive coefficients in urban populations but negative coefficients in nonurban populations (Figure S4). However, only one of the four negative correlation coefficients reported had confidence intervals that did not overlap zero (Caizergues et al., 2022). Urbanization did not appear to predict strength of correlations: while 8 paired comparisons had stronger correlations in urban populations than in nonurban populations, 6 had stronger correlations in nonurban populations (Figure S4).

### 3.5 Effects of moderators

For SMD, effect sizes for all moderators were positive (Figure S5, Table 2). Specifically, urban populations exhibited increased average behaviours relative to nonurban populations regardless of taxonomic class, ecological category or urban tolerance category, though confidence intervals overlapped zero in many cases, particularly in categories with few samples. For example, animals with ectothermic thermoregulation were poorly represented in the dataset, reflecting the overarching taxonomic bias (*k* = 24; 4 amphibians, 8 insects, 12 reptiles; Table 2). Similarly, nocturnal (*k* = 18) and cathemeral species (*k* = 17) were underrepresented, as were effect sizes from common‐garden experiments (*k* = 8). In these underrepresented moderator categories, meta‐analytic estimates lacked precision: Even when effect sizes appeared biologically meaningful, confidence intervals were broad, indicating low statistical power and high uncertainty in the estimates (Figure S5, Table 2). Interestingly, species with more ‘generalist’ characteristics (i.e. those filling broader ecological niches, Rivkin et al., 2019) did not seem to have behavioural responses that differed from specialist species (Figure S5, Table 2).

In contrast, we did not identify consistent patterns of differences in phenotypic variation (∆ln(SD)) among moderator categories (Figure S5, Table 2). Across all moderators, only two estimates showed significant differences in phenotypic variation between urban and nonurban populations: first, ‘avoider’ species exhibited significantly less phenotypic variation in urban populations relative to nonurban populations (∆ln(SD) [95% HDPI]: −0.48 [−0.89, −0.10]); second, estimates from wild‐caught animals tested in captivity were slightly higher in urban populations (0.24 [0.06, 0.43]). No other moderator categories showed statistically significant differences, indicating a lack of consistent, biologically meaningful patterns across moderators.

### 3.6 Heterogeneity

Total heterogeneity was high (>75%) for all models (Table S4). Study‐level heterogeneity ranged from low (<15%) to high (>75%). Studies reporting behavioural syndromes had the lowest inter‐study heterogeneity (12.5%), and studies reporting SMD had the greatest inter‐study heterogeneity (75%). Heterogeneity due to phylogenetic or species‐level effects ranged from 0% to 18%, indicating a non‐negligible but weak phylogenetic signal. Research group was not included as a random effect due to ambiguity in group assignment but is a potential additional contributor to heterogeneity.

### 3.7 Assessment for publication bias

We investigated the presence of small‐study and time‐lag effects publication biases. Funnel plots were asymmetric, indicating publication bias (Figure S7); however, multilevel meta‐regression analysis for publication bias did not reveal strong evidence for publication bias related to either time lag (β [95% CI]; $\beta_{\text{Year}}$ = −0.26 [−0.64, 0.10]) or study size ($\beta_{\mathrm{SE}}$ = 0.48 [−0.15, 1.12]).

## 4 DISCUSSION

In this study, we used phylogenetically controlled meta‐analyses to examine urban‐associated behavioural differences in wild animals, focusing on four behaviours: aggressiveness, boldness, exploration and activity. We compared urban and nonurban average behavioural traits, phenotypic variation, behavioural repeatability and correlations. While we found no evidence that urbanization was clearly associated with differences in behavioural variation, repeatability or behavioural correlations, we found that urban populations were on average bolder, more aggressive, more explorative and more active relative to nonurban conspecifics, with boldness showing the strongest urban‐associated increases (Figure 2, Table 1). These findings were robust in avian species, which were well represented in the studies we reviewed (72.0% of all estimates; Figure 1a). Conversely, among non‐avian species—which were considerably less well represented across studies—boldness was the only behaviour that was significantly increased in urban populations, though non‐avian urban populations tended to be more aggressive than their nonurban counterparts (Table 1). Surprisingly, urban‐associated increases in behavioural traits occurred across species irrespective of ecological category (Table 2). Overall, our findings of mean differences suggest the possibility that on a global scale, urban populations might experience directional selection on ecologically relevant behavioural phenotypes. Our findings also highlight key research opportunities to improve our ability to draw generalizable conclusions across a broader range of phenotypes and taxa, particularly ectothermic and non‐avian species. Such efforts may help uncover patterns that could otherwise go unnoticed.

### 4.1 Urbanization is associated with behavioural shifts in wild populations

Urbanization has been extensively linked to shifts in mean phenotypes, including shifts in morphology (e.g. limb structure in Anolis lizards, Winchell et al., 2023; melanism in peppered moths, Kettlewell, 1958, 1956), life‐history strategies (e.g. clutch size and lay date in birds, (Capilla‐Lasheras et al., 2022; Sepp et al., 2018)) and physiology (e.g. thermal tolerance in ants, Diamond et al., 2017; stress response in juncos, Atwell et al., 2012). Here, we found compelling quantitative evidence that urbanization is also linked to shifts in mean behavioural traits (Figure 2a). These results expand our understanding of urban‐associated phenotypic shifts and complement findings from previous reviews (Lowry et al., 2013; Putman & Tippie, 2020; Ritzel & Gallo, 2020; Sol et al., 2013). Urban populations showed increases in all four behavioural traits, particularly boldness and aggressiveness, though the magnitude of this response varied across taxa. When we considered only avian taxa, urban populations showed strong and statistically significant increases in all four behavioural traits (note, however, that results for activity were based only on one paired urban–nonurban effect size, Table 1).

Conversely, directional urban‐specific behavioural differences were less evident among non‐avian species: boldness was the sole behaviour expressing a significant increase in urban populations, and no other behaviours differed statistically between urban and nonurban populations, though urban populations tended to be more aggressive (Table 1). While the lack of significant behavioural change in non‐avian species could indicate a true absence of urban behavioural change, that is, a biologically meaningful non‐result, an alternative explanation is that a limited sample size reduced our ability to detect nonurban–urban differences in non‐avian species. Indeed, though we strove to expand on previous work in birds by including estimates from mammals, amphibians, reptiles and invertebrates, these non‐avian species were poorly represented in our final dataset (Figure 1a), reflecting ongoing taxonomic representation issues widely observed in animal behaviour and biodiversity research (Rosenthal et al., 2017; Troudet et al., 2017). This taxonomic bias has potentially profound implications for meta‐analyses (Dochtermann et al., 2026).

Additionally, our analyses relied on a binary urban–nonurban classification, which simplifies the complex and continuous nature of urbanization. While this approach allowed for a standardized comparison across studies, it may obscure subtle behavioural responses along gradients of urban intensity, such as variation in impervious cover or population density, and should be considered a limitation of our study. Unfortunately, due to variation among studies in how urbanization was measured and reported, this binary classification was not avoidable. Moreover, when reported, continuous measures of urbanization were typically used to describe population conditions. Future studies should consider finer‐scale measurement of behaviours along gradients.

Boldness was substantially higher in urban populations, a robust pattern observed across both non‐avian and avian taxa. Several factors could contribute to this phenomenon. First, being bolder and taking more risks may increase an individual's likelihood of invading urban areas, either because bold individuals have a greater propensity for dispersal, either actively (Bégué et al., 2023; Dingemanse et al., 2003; Fraser et al., 2001) or passively (e.g. ‘uptake’; Chapple et al., 2012); or because bolder animals prefer specific habitat characteristics that may be present in an urban environment (e.g. personality‐matching habitat choice; Holtmann et al., 2017; Schirmer et al., 2019). Alternatively, though not mutually exclusively, higher boldness may be an adaptive plastic or genetic response that increases the likelihood of settling or surviving in urban habitats. Increased boldness may give individuals a competitive edge in urban environments with limited or low‐quality resources (Clermont et al., 2023; Tremmel & Müller, 2013) or changed predator communities (Ward‐Fear et al., 2018). Bolder animals may also be less sensitive to human presence (McGiffin et al., 2013; Uchida et al., 2019).

It is worth noting that in the analysed dataset, flight initiation distance (FID) studies comprised 84.2% of all estimates of boldness; the lack of sufficient representation for other behavioural assays thus prevented us from disentangling increased human tolerance from increased risk assessment. While FID is a popular field measure commonly used as a proxy for risk‐taking in avian species and other taxa (Stankowich & Blumstein, 2005), the biological significance of this metric in response to humans, rather than non‐human predators, is uncertain. The prevalence of measures of FID in avian studies in our dataset suggests that we may be capturing human tolerance more than risk assessment in birds, though several studies in birds have linked FID to various ecological conditions and fitness‐related traits relevant to risk‐taking behaviour (Møller, 2014; Møller & Garamszegi, 2012). Studies validating the biological significance of FID and other behavioural metrics are clearly needed to clarify what exactly these metrics capture and their ecological relevance (e.g. Carter et al., 2013; Dumont et al., 2012; Perals et al., 2017). In addition, more studies are needed to help disentangle the drivers of urban‐associated boldness. Finally, future work incorporating migration‐dispersal dynamics and assessment of fitness benefits of behavioural responses will yield valuable insights on the causes and consequences of increased boldness in urban populations.

### 4.2 No evidence for differences in behavioural variation

We did not find evidence that urban and nonurban populations differed in behavioural variation (∆ln(SD), Figure 2b; Table 1). This result contrasts with findings from two recent meta‐analyses reporting urban‐associated increased phenotypic variation in a variety of life history and morphological traits populations (Capilla‐Lasheras et al., 2022; Thompson et al., 2022) but is consistent with a large‐scale study demonstrating a lack of clear patterns. Specifically, in a large‐scale analysis of phenotypic variation spanning 4507 effect sizes, 196 studies and 177 species, Sanderson and colleagues found that phenotypic variation did not differ in a generalizable way by either trait type or human disturbance (Sanderson et al., 2023). Their results suggest that there may not be a universal pattern in how phenotypic variation responds to environmental conditions.

Importantly, a lack of consistent differences does not imply that populations are not changing or evolving; rather, it may reflect the influence of context‐dependent factors such as ecological interactions, evolutionary histories and the timeline of urban colonization. For example, if some urban populations are well established in their local environments, and have thus already modified fitness‐related behaviours through plasticity or adaptation, we might expect selection to have already reduced variation in those traits. In contrast, populations in the early stages of urban colonization may still be undergoing rapid shifts in trait means and variances. Thus, heterogeneity in evolutionary stage and environmental context across studies could obscure broader patterns. Moving forward, research should consider the evolutionary stage of urban populations when assessing phenotypic variation, as this could provide key insights into how variation is shaped by both urbanization and selection pressures. Even in the absence of general patterns, it will still be valuable to explore how phenotypic variation responds to urbanization within specific contexts (e.g. particular species, phenotypes or urban characteristics) to better understand the eco‐evolutionary dynamics underlying urban‐associated phenotypic change.

### 4.3 Implications for behavioural plasticity and evolutionary constraints

Urban and nonurban populations did not differ in repeatability, that is, the amount of phenotypic variance attributable to among‐individual variance (Figure 3). Instead, we found evidence that in both urban and nonurban environments, the repeatability of each behaviour ranged from low (*τ* = 0.19) to moderately high (*τ* = 0.50), with boldness being the behaviour with the highest repeatability. These values fall within the ranges of previously reported repeatabilities for ‘personality’‐related behaviours (average *τ*, range; Dochtermann et al., 2015: 0.62 (0.37–0.83); Bell et al., 2009: 0.37 (−0.22–0.97)). One notable difference in our study, however, is that aggressiveness fell on the lower side of this range for both urban and nonurban populations (urban *τ* = 0.21, nonurban *τ* = 0.19), in contrast to previous reports of high repeatability for aggressiveness (Dochtermann et al., 2015, *τ* = 0.65; Bell et al., 2009: *τ* = 0.48). This contradictory finding could be explained by the fact that in a focal individual, aggressive behaviour is highly dependent on the social context (in particular, an opponent) and often influenced by indirect genetic effects (e.g. Bailey et al., 2018; Moore et al., 1997; Wolf et al., 1998). Alternatively, since we independently assigned behaviours to categories, prior meta‐analyses and individual studies may have conflated multiple different behaviours under the aggressiveness label. For example, aggressiveness is sometimes defined as agonistic interactions with conspecifics (Oyegbile & Marler, 2005), heterospecifics (Vullioud et al., 2013) or humans (Class et al., 2014), and these behaviours are not necessarily equivalent (Gupta et al., 2019; Peiman & Robinson, 2010).

Given that repeatabilities provide an upper limit for heritability, our results imply that both urban and nonurban populations likely retain heritable variation on which selection can act. This is a very tentative conclusion, however, for three reasons: (1) There were very few studies that reported repeatability; (2) differences in repeatability are difficult to interpret given that they may differ due to divergence in both among‐ and within‐individual variances (Dochtermann & Royauté, 2019; Royauté & Dochtermann, 2021; Wilson, 2018); and (3) repeatability is not informative as to a lower limit of heritability. Additionally, variation in behavioural assay type may contribute to heterogeneity in repeatability estimates, but current sample sizes are too limited to evaluate this systematically. Consequently, future research should prioritize the reporting of the variance components that contribute to repeatabilties, the estimation of additive genetic variation, and the use of multiple, comparable assays where feasible. For similar reasons, the potential for behavioural correlations to constrain evolutionary responses to differing urban and nonurban selective regimes remains unclear.

## 5 CONCLUSION

Urban evolutionary ecology is a rapidly growing field, motivated by the urgent need to understand how organisms respond to anthropogenic pressures in an era of rapid environmental change. Our study highlights several key limitations in the existing literature that constrain broad inference and interpretation; addressing these gaps will be essential for understanding how selection operates in urban environments and for understanding species' vulnerability to the Anthropocene.

First, taxonomic biases in the available literature restricted our ability to perform robust sub‐group analyses. Amphibians, reptiles and insects were each represented by fewer than a dozen studies; by comparison, birds were represented by 201 effect sizes. This imbalance reduces our ability to determine whether patterns observed in birds are representative of other animals more broadly. Future work should aim to include a wider range of species, particularly non‐avian ectotherms and, especially, invertebrates (Dochtermann et al., 2026).

Second, future work should more explicitly examine how natural selection in urban environments might drive the phenotypic changes observed in urban populations, and importantly, distinguish between genetic adaptations and plastic responses. This question can be addressed using conventional eco‐evolutionary approaches such as common‐garden experiments, estimates of selection coefficients, genetic sampling and genome‐wide association studies (Charmantier et al., 2024; Lambert et al., 2021; Thompson et al., 2022). In our review, only eight studies employed common‐garden designs; however, because we did not explicitly search for studies using genomic methods, we cannot evaluate how frequently such approaches have been used. Without targeted investigations, it remains unclear whether behavioural differences between urban and nonurban populations reflect heritable change or short‐term plasticity. Addressing this gap will require experimental or longitudinal approaches that explicitly test for genetically based behavioural responses to urban pressures.

Finally, behavioural data were unevenly distributed across activity patterns. Nocturnal species remain particularly understudied, likely due to logistical challenges in observation. However, these species may be among the most strongly affected by anthropogenic light and nighttime human activity. Increased attention to nocturnal, crepuscular and cathemeral taxa would improve understanding of how diel patterns mediate behavioural adaptation to urban environments.

In summary, our results show that urbanization is associated with substantial changes to behaviour. Our results also highlight that these changes are pervasive across geographic regions, ecological niche and species. Future research determining whether urban‐related behaviours represent adaptations to urban environments will help clarify how selection has changed in the Anthropocene and to what degree organisms can respond to these changes.

## AUTHOR CONTRIBUTIONS

Tracy T. Burkhard conceived the study, conducted the literature search, extracted data, performed analyses and wrote the original draft. Anne Charmantier extracted data and supervised the project. Ned A. Dochtermann contributed to study design, performed analyses and supervised statistical work. All authors contributed to the preregistration, study design and review and editing of the manuscript.

## FUNDING INFORMATION

This work was supported by an NSF Postdoctoral Research Fellowship in Biology to T.T.B.

## CONFLICT OF INTEREST STATEMENT

There are no conflicts of interest.

## ETHICAL APPROVAL

As a meta‐analysis of previously published data, this article does not present data requiring ethics board approval.

## STATEMENT ON INCLUSION

This study is a global meta‐analysis based entirely on previously published data; no new local data collection was conducted. However, the dataset incorporates studies from institutions across every continent except Antarctica. The project itself represents an international collaboration among an American postdoctoral researcher/new professor formerly based in France, now in Portland, Oregon (TTB) and two senior researchers based in France and the United States (AC and NAD), fostering new cross‐continental collaboration.

## Supporting information


**Figure S1.** Prisma diagram.
**Figure S2.** Orchard plot for SMD. Raw data points plotted alongside meta‐analytic estimates shown with 95% confidence intervals. Size of individual data points represents precision (inverse of standard error).
**Figure S3.** Orchard plot for ∆ln(SD). Raw data points plotted alongside meta‐analytic estimates shown with 95% highest density posterior intervals (HDPI). Size of individual data points represents precision (inverse of standard error).
**Figure S4.** Forest plot of behavioral correlation coefficients for urban and nonurban populations from individual studies included in the meta‐analysis. Black error bars indicate 95% confidence intervals; *N* provides the number of individuals sampled in each population. Study authors and year of publication, taxa clade, species, and population provided.
**Figure S5.** Moderator forest plots of estimates for standardized mean differences, SMD. Positive values on the *x*‐axis represent higher mean in urban populations than in nonurban populations. Black error bars indicate 95% confidence intervals; *k* indicates the number of paired urban‐nonurban observations.
**Figure S6.** Moderator forest plots of estimates for log standard deviation, ∆ln(SD). Positive values on the x‐axis represent higher variation in urban populations than in nonurban populations. Black error bars indicate 95% credible intervals calculated from highest posterior density interval (HDPI); *k* indicates the number of paired urban‐nonurban observations.
**Figure S7.** Funnel plots used to estimate publication bias in the full datasets for (A) SMD, (B) repeatability, and (C) behavioral syndromes. Meta‐analytic means are indicated with dashed lines.
**Table S1.** List of deduplicated studies.
**Table S2.** Estimates of repeatability with associated 95% confidence intervals. Estimates have been back‐transformed from Fisher's *z* estimates. *K* indicates the number of paired urban‐nonurban observations for each moderator.
**Table S3.** Estimates of behavioral correlations, *r*, with associated 95% confidence intervals. Estimates have been back‐transformed from Fisher's *z* estimates. *K* indicates the number of paired urban‐nonurban observations for each moderator.
**Table S4.** Heterogeneity estimates for meta‐analytic models.

## Data Availability

Data are available as online Supporting Information.

## APPENDIX A

Data for this study are available in the Supporting Information.

We performed the following searches: (**1**) Topic = [(*list of taxa*) AND (*list of urban habitats*) AND (*list of nonurban habitats*) AND (*list of behaviors*) AND (animal wild)]; (**2**) Topic = [(*list of taxa*) AND (*list of urban habitats*) AND (*list of nonurban habitats*) AND (*list of behaviors*)] AND Research Areas = (Zoology, Behavioral Sciences, Ornithology, Evolutionary Biology, Entomology); (**3**) Topic = [(*list of taxa*) AND (*list of urban habitats*) AND (*list of nonurban habitats*) AND (*list of tests*)]; (**4**) Topic = [(*list of taxa*) AND (*list of urban habitats*) AND (*list of nonurban habitats*) AND (behavioral syndrome OR behavioural syndrome OR Bold* OR Explorat* OR Neophilia OR Neophobia OR Novel object)]; (**5**) Topic = [(*list of taxa*) AND (*list of urban habitats*) AND (*list of nonurban habitats*) AND (proactive NEAR/1 reactive) syndrome OR personality traits OR (pace of life NEAR/1 behavior)].

Each ‘*list’* search term contained a list of the keywords used to specify taxa, environment and behaviour or behavioural assay:

*list of taxa* = (mammal* OR wild OR animal OR bird OR vertebrate OR avian OR reptil* OR herp* OR amphibian OR anuran OR frog OR lizard OR insect OR arthropod OR spider OR snake OR invertebrate).

*list of urban habitats* = (anthropogenic disturbance OR human disturbance OR urban* OR city* OR town* OR metro*).

*list of nonurban habitats* = (rural OR farm* OR agricult* OR country OR countryside OR natural environment OR forest*).

*list of behaviors* = (affiliative OR affiliat* OR aggress* OR alarm OR anti‐predator OR behavioural syndrome OR behavioral syndrome OR bold* OR defense OR escape OR explor* OR fear OR FID OR flight initiation distance OR gregarious* OR neophilia OR neophobia OR personality OR risk‐taking OR shy OR sociability OR temperament OR vigilance).

*list of tests* = (open field OR novel object OR handling bag test OR hole board test OR (light NEAR/1 dark) test OR light–dark test OR predator presentation test OR predator stimulus test OR novel environment OR mirror image OR mirror test OR attack latency OR dyadic encounter OR diadic encounter).
