# Supplementary material for: Global meta‐analysis reveals urban‐associated behavioural differences among wild populations
Source: J Anim Ecol. 2026 May 18;95(9):1539–57. doi: 10.1111/1365-2656.70269 (PMC13536287; doi:10.1111/1365-2656.70269)
Supplement: Supplementary file 1 [file JANE-95-1539-s001.zip › Supplements for Burkhard et al 2026/Burkhard_etal_2026_Supplemental Information.docx]

*Supplementary materials for:*

Global meta-analysis reveals urban-associated behavioral differences among wild populations

***Authors****:* Tracy T. Burkhard (ORCID: 0000-0002-1013-2782)^1,2,†^, Ned A. Dochtermann (ORCID: 0000-0002-8370-4614)^3^, Anne Charmantier (ORCID: 0000-0002-0691-2647)^1^

^1^ Centre d’Ecologie Fonctionnelle et Evolutive, Université de Montpellier, CNRS, EPHE, IRD, Montpellier, France

^2^ Institut des Sciences de l’Evolution de Montpellier, Université de Montpellier, CNRS, IRD, Montpellier, France

^3^ Department of Biological Sciences, North Dakota State University, Fargo, North Dakota, USA

^†^ Present address: Lewis & Clark College, Portland, Oregon, USA

***Corresponding author:***

Tracy Tran Burkhard

615 S Palatine Hill Rd

Portland, OR 97219

949 302-9277; [tburkhard@lclark.edu](mailto:tburkhard@lclark.edu)

***Key words:*** Urbanization, animal personality, behavioral change, repeatability, phenotypic plasticity, behavioral flexibility, behavioral syndrome, functional traits

# Supplemental Methods

## Deviations from preregistration

Our final meta-analysis deviated from the preregistration in several ways:

- First, we could not specify certain analyses beforehand due to the uncertainty regarding the available data and its scope.
- For studies comparing multiple populations along an urban gradient, we included estimates only for the most urban and least urban populations to maintain consistency.
- We were also unable to use degree of urbanization as a moderator because studies differed substantially in both the method used to assess urbanization and in whether they reported this method or measurement.
- We were unable to fit behavioral correlation-specific models for syndromes due to the insufficient number of studies.
- The limited number of studies on non-avian taxa restricted our ability to conduct separate analyses for each taxonomic class.

## Supplementary literature search in SCOPUS

Following the penultimate round of peer review, we conducted supplemental literature searches to assess whether our restriction to Web of Science (WoS) might have systematically excluded relevant studies. While WoS and SCOPUS are known to have largely overlapping coverage in ecology and evolutionary biology, by contrast, PubMed is primarily focused on biomedical research and would be unlikely to contribute substantially to studies on wildlife behavioral ecology. Therefore, we opted to conduct our supplemental searches in SCOPUS.

We conducted three searches in SCOPUS on February 28, 2026. Searches were designed to replicate our original strategy using WoS as closely as possible, while accounting for differences in syntax, subject area categories, and search operators. We focused on the main search—studies assessing animal behavior in relation to urbanization and human disturbance—and on keywords related to behavioral repeatability, as suggested in an earlier round of peer review. We did not replicate all “behavioral test” searches (*e.g.,* open-field test, light-dark test) due to the substantial time required for screening studies for inclusion.

Because none of the authors’ home institutions have subscriptions to SCOPUS, a limitation that informed our preregistered study design, we borrowed access through a neighboring institution to conduct the supplemental search. This underscores a broader challenge for meta-analysts at smaller institutions or in resource-limited settings, where access to multiple databases may not be feasible.

Our SCOPUS searches returned 213 results, of which 89 were duplicates, leaving 124 unique papers. One study was excluded because its publication date (December 2022) was after our original search (September 2022), thus leaving 123 papers. Of these, 59 had already been captured in our WoS searches, an overlap of 48%. Screening the remaining 64 studies according to our inclusion criteria identified a single additional eligible study:

Daniel, A., & Chaves-Campos, J. (2021). Contrary to vertebrates, less aggressive and more consistent individuals are common in disturbed habitats in the colonial spider *Metabus gravidus* (Araneae: Araneidae). *Behaviour*, *158*(3-4), 225-248. <https://doi.org/10.1163/1568539X-bja10063>

Daniel & Chaves-Campos (2021) provide measures of behavior and behavioral repeatability in an invertebrate; while this study would have been valuable to include, its singular addition suggests that differences in database coverage are minimal and would not meaningfully alter the patterns observed in our meta-analysis. Based on these results, we are confident that despite practical limitations in database access, restricting our search to WoS did not meaningfully affect the comprehensiveness or taxonomic breadth of our dataset.

### Search terms

Search 1: 28 Feb 2026. 125 results

TITLE-ABS-KEY( mammal* OR "wild animal" OR bird OR vertebrate OR avian OR reptil* OR herp* OR amphibian OR anuran OR frog OR lizard OR insect OR arthropod OR spider OR snake OR invertebrate) AND TITLE-ABS-KEY("anthropogenic disturbance" OR "human disturbance" OR urban* OR city* OR town* OR metro*) AND TITLE-ABS-KEY(wild animal) AND TITLE-ABS-KEY(rural OR farm* OR agricult* OR country OR countryside OR "natural environment" OR forest*) AND TITLE-ABS-KEY(affiliative OR affiliat* OR aggress* OR alarm OR "anti-predator" OR "behavioural syndrome" OR "behavioral syndrome" OR bold* OR defense OR escape OR explor* OR fear OR FID OR "flight initiation distance" OR gregarious* OR neophilia OR neophobia OR personality OR "risk-taking" OR shy OR sociability OR temperament OR vigilance) AND PUBYEAR > 1955 AND PUBYEAR < 2023 AND ( LIMIT-TO ( DOCTYPE,"ar" ) )

Search 2: 5 results

TITLE-ABS-KEY ( mammal* OR "wild animal" OR bird OR vertebrate OR avian OR reptil* OR herp* OR amphibian OR anuran OR frog OR lizard OR insect OR arthropod OR spider OR snake OR invertebrate ) AND TITLE-ABS-KEY ( "anthropogenic disturbance" OR "human disturbance" OR urban* OR city* OR town* OR metro* ) AND TITLE-ABS-KEY ( wild animal ) AND TITLE-ABS-KEY ( rural OR farm* OR agricult* OR country OR countryside OR "natural environment" OR forest* ) AND TITLE-ABS-KEY ( affiliative OR affiliat* OR aggress* OR alarm OR "anti-predator" OR "behavioural syndrome" OR "behavioral syndrome" OR bold* OR defense OR escape OR explor* OR fear OR FID OR "flight initiation distance" OR gregarious* OR neophilia OR neophobia OR personality OR "risk-taking" OR shy OR sociability OR temperament OR vigilance ) AND TITLE-ABS-KEY ( repeatab* OR "among individual variation" OR "between individual variation" OR "among-individual differences" OR "individual behavioral consistency" OR "individual behavioural consistency" OR intra-individual OR inter-individual ) AND PUBYEAR > 1955 AND PUBYEAR < 2023 AND ( LIMIT-TO ( DOCTYPE , "ar" ) )

Search 3: 28 Feb 2026. 83 results

TITLE-ABS-KEY( mammal* OR "wild animal" OR bird OR vertebrate OR avian OR reptil* OR herp* OR amphibian OR anuran OR frog OR lizard OR insect OR arthropod OR spider OR snake OR invertebrate) AND TITLE-ABS-KEY("anthropogenic disturbance" OR "human disturbance" OR urban* OR city* OR town* OR metro*) AND TITLE-ABS-KEY(wild animal) AND TITLE-ABS-KEY(rural OR farm* OR agricult* OR country OR countryside OR "natural environment" OR forest*) AND TITLE-ABS-KEY(affiliative OR affiliat* OR aggress* OR alarm OR "anti-predator" OR "behavioural syndrome" OR "behavioral syndrome" OR bold* OR defense OR escape OR explor* OR fear OR FID OR "flight initiation distance" OR gregarious* OR neophilia OR neophobia OR personality OR "risk-taking" OR shy OR sociability OR temperament OR vigilance) AND PUBYEAR > 1955 AND PUBYEAR < 2023 AND ( LIMIT-TO ( DOCTYPE,"ar" ) AND ( LIMIT-TO ( SUBJAREA,"ENVI" ) OR LIMIT-TO ( SUBJAREA,"AGRI" ) )

## Bayesian meta-regressions

For analyses comparing ∆ln(SD), we used the MCMCglmm package (version 2.34, Hadfield, 2010). We ran each model for 8,000,000 iterations with burn-in of 6,000,000 and selected a thinning interval of 2000 to reduce autocorrelation. This resulted in a posterior distribution sample size of 1000. For each model, we visually checked chains for convergence and mixing. We provided each model with an inverse gamma prior for random effects (V = 1, nu = 0.002) and used the default non-informative (flat) prior for fixed effects. We included population as a fixed effect, then calculated the difference between the urban and nonurban posterior distributions using the *describe_posterior* function from the bayestestR package (version 0.13.0, Makowski et al., 2019). To account for the mean-variance relationship, we also included the log mean as a fixed effect (Nakagawa et al., 2015). Inference on differences were based on posterior estimates and credible intervals (*i.e.*, highest posterior density intervals) overlapping zero or not. We then assessed urban effects per behavior and accounted for behavior-specific means and variances and possible correlations among behaviors by fitting a multivariate meta-analytic model with an unstructured variance-covariance matrix.

# Supplementary figures

## Figure S1

Prisma diagram

## Figure S2

Orchard plot for SMD. Raw data points plotted alongside meta-analytic estimates shown with 95% confidence intervals. Size of individual data points represents precision (inverse of standard error).

## Figure S3

Orchard plot for ∆ln(SD). Raw data points plotted alongside meta-analytic estimates shown with 95% highest density posterior intervals (HDPI). Size of individual data points represents precision (inverse of standard error).

## Figure S4

Forest plot of behavioral correlation coefficients for urban and nonurban populations from individual studies included in the meta-analysis. Black error bars indicate 95% confidence intervals; N provides the number of individuals sampled in each population. Study authors and year of publication, taxa clade, species, and population provided.

## Figure S5

Moderator forest plots of estimates for standardized mean differences, SMD. Positive values on the x-axis represent higher mean in urban populations than in nonurban populations. Black error bars indicate 95% confidence intervals; *k* indicates the number of paired urban-nonurban observations.

## Figure S6

Moderator forest plots of estimates for log standard deviation, ∆ln(SD). Positive values on the x-axis represent higher variation in urban populations than in nonurban populations. Black error bars indicate 95% credible intervals calculated from highest posterior density interval (HDPI); *k* indicates the number of paired urban-nonurban observations.

## Figure S7

Funnel plots used to estimate publication bias in the full datasets for (A) SMD, (B) repeatability, and (C) behavioral syndromes. Meta-analytic means are indicated with dashed lines.

# Supplementary tables

## Table S1

List of deduplicated studies. (See external excel file)

## Table S2

Estimates of repeatability with associated 95% confidence intervals. Estimates have been back-transformed from Fisher’s *z* estimates. *K* indicates the number of paired urban-nonurban observations for each moderator.

| Category | Population | *k* | *τ* [95% CI] |
| --- | --- | --- | --- |
| Intercept-only | Urban | 29 | 0.41 [0.29, 0.51] |
|  | Nonurban |  | 0.36 [0.24, 0.47] |
| Activity | Urban | 3 | 0.44 [0.18, 0.64] |
|  | Nonurban |  | 0.35 [0.07, 0.57] |
| Aggressiveness | Urban | 2 | 0.21 [-0.14, 0.51] |
|  | Nonurban |  | 0.19 [-0.17, 0.51] |
| Boldness | Urban | 10 | 0.49 [0.33, 0.63] |
|  | Nonurban |  | 0.50 [0.33, 0.64] |
| Exploration | Urban | 14 | 0.35 [0.20, 0.49] |
|  | Nonurban |  | 0.28 [0.12, 0.42] |

## Table S3

Estimates of behavioral correlations, *r*, with associated 95% confidence intervals. Estimates have been back-transformed from Fisher’s *z* estimates. *K* indicates the number of paired urban-nonurban observations for each moderator.

| Category | Population | *k* | *r* [95% CI] |
| --- | --- | --- | --- |
| Intercept-only | Urban | 14 | 0.32 [0.13, 0.49] |
|  | Nonurban |  | 0.34 [0.15, 0.50] |
| Boldness and exploration | Urban | 4 | 0.18 [-0.10, 0.43] |
|  | Nonurban |  | 0.06 [-0.22, 0.33] |
| Activity and boldness | Urban | 3 | 0.51 [0.23, 0.71] |
|  | Nonurban |  | 0.41 [0.11, 0.64] |
| Activity and exploration | Urban | 1 | 0.63 [0.22, 0.86] |
|  | Nonurban |  | -0.09 [-0.55, 0.40] |
| Aggression and boldness | Urban | 6 | 0.18 [-0.15, 0.47] |
|  | Nonurban |  | 0.57 [0.30, 0.75] |

## Table S4

Heterogeneity estimates for meta-analytic models.

| Effect size | Descriptor | I2 Total | I2 study id | I2 obs id | I2 scientific name phylo | I2 scientific name |
| --- | --- | --- | --- | --- | --- | --- |
| SMD | Global, intercept-only | 98.41 | 75.10 | 9.30 | 14.01 | 0.00 |
| ∆ln(SD) | Global, intercept-only | 99.73 | 78.83 | 20.82 | 0.003 | 0.004 |
| Syndrome | Global, intercept-only | 84.76 | 11.11 | 73.65 | 0.00 | 0.00 |
| Repeatability | Global, intercept-only | 75.82 | 22.91 | 33.76 | 1.39 | 17.76 |
|  |  |  |  |  |  |  |

# References

Blair, R. B. (1996). Land use and avian species diversity along an urban gradient. *Ecological Applications*, *6*(2), 506–519. https://doi.org/10.2307/2269387

Bonier, F., Martin, P. R., & Wingfield, J. C. (2014). *Urban birds have broader environmental tolerance*. *January 2008*. https://doi.org/10.1098/rsbl.2007.0349

Croci, S., Butet, A., & Clergeau, P. (2008). Does urbanization filter birds on the basis of their biological traits? *Condor*, *110*(2), 223–240. https://doi.org/10.1525/cond.2008.8409

Digweed, S. C. (1994). The Coleopterists Society Detection of Mucus-Producing Prey by Carabus nemoralis Mueller and Scaphinotus marginatus. *Coleopterists Bulletin*, *48*(4), 361–369.

Fischer, J. D., Schneider, S. C., Ahlers, A. A., & Miller, J. R. (2015). Categorizing wildlife responses to urbanization and conservation implications of terminology. *Conservation Biology*, *29*(4), 1246–1248. https://doi.org/10.1111/cobi.12451

Koolhaas, J. M., Korte, S. M., De Boer, S. F., Van Der Vegt, B. J., Van Reenen, C. G., Hopster, H., De Jong, I. C., Ruis, M. A. W., & Blokhuis, H. J. (1999). Coping styles in animals: Current status in behavior and stress- physiology. *Neuroscience and Biobehavioral Reviews*, *23*(7), 925–935. https://doi.org/10.1016/S0149-7634(99)00026-3

Luff, M. L. (1978). Diel activity patterns of some field Carabidae. *Ecological Entomology*, *3*(1), 53–62. https://doi.org/10.1111/j.1365-2311.1978.tb00902.x

McKinney, M. L. (2002). Urbanization, biodiversity, and conservation. *BioScience*, *52*(10).

Meiri, S. (2018). Traits of lizards of the world: Variation around a successful evolutionary design. *Global Ecology and Biogeography*, *27*(10), 1168–1172. https://doi.org/10.1111/geb.12773

Meiri, S. (2019). Data from: Traits of lizards of the world: variation around a successful evolutionary design. *Dryad Dataset*.

Melliger, R. L., Braschler, B., Rusterholz, H. P., & Baur, B. (2018). Diverse effects of degree of urbanisation and forest size on species richness and functional diversity of plants, and ground surface-active ants and spiders. *PLoS ONE*, *13*(6), 1–24. https://doi.org/10.1371/journal.pone.0199245

Moll, R. J., Cepek, J. D., Lorch, P. D., Dennis, P. M., Robison, T., & Montgomery, R. A. (2020). At what spatial scale(s) do mammals respond to urbanization? *Ecography*, *43*(2), 171–183. https://doi.org/10.1111/ecog.04762

Moll, R. J., Cepek, J. D., Lorch, P. D., Dennis, P. M., Tans, E., Robison, T., Millspaugh, J. J., & Montgomery, R. A. (2019). What does urbanization actually mean? A framework for urban metrics in wildlife research. *Journal of Applied Ecology*, *56*(5), 1289–1300. https://doi.org/10.1111/1365-2664.13358

Oliveira, B., Sao-Pedro, V., Santos-Barrera, G., Penone, C., & C. Costa, G. (2017). *AmphiBIO_v1*. 10.6084/m9.figshare.4644424.v5.

Oliveira, B., Sao-Pedro, V., Santos-Barrera, G., Penone, C., & Costa, G. (2017). AmphiBIO, a global database for amphibian ecological traits. *Scientific Data*. https://doi.org/10.1038/sdata.2017.123.

Réale, D., Garant, D., Humphries, M. M., Bergeron, P., Careau, V., & Montiglio, P. O. (2010). Personality and the emergence of the pace-of-life syndrome concept at the population level. In *Philosophical Transactions of the Royal Society B: Biological Sciences* (Vol. 365, Issue 1560, pp. 4051–4063). https://doi.org/10.1098/rstb.2010.0208

Réale, D., Reader, S. M., Sol, D., McDougall, P. T., & Dingemanse, N. J. (2007). Integrating animal temperament within ecology and evolution. *Biological Reviews*, *82*(2), 291–318. https://doi.org/10.1111/j.1469-185X.2007.00010.x

Rodriguez, J. T., Lesmeister, D. B., & Levi, T. (2021). *Mesocarnivore landscape use along a gradient of urban , rural , and forest cover*. 1–25. https://doi.org/10.7717/peerj.11083

RS-eco. (2022). *traitdata: Easy access to various ecological trait data* (R package version 0.0.1).

Santini, L., González-Suárez, M., Russo, D., Gonzalez-Voyer, A., von Hardenberg, A., & Ancillotto, L. (2019a). One strategy does not fit all: determinants of urban adaptation in mammals. *Ecology Letters*, *22*(2), 365–376. https://doi.org/10.1111/ele.13199

Santini, L., González-Suárez, M., Russo, D., Gonzalez-Voyer, A., von Hardenberg, A., & Ancillotto, L. (2019b). One strategy does not fit all: determinants of urban adaptation in mammals. *Ecology Letters*, *22*(2), 365–376. https://doi.org/10.1111/ele.13199

Smith, B. R., & Blumstein, D. T. (2008). Fitness consequences of personality: A meta-analysis. *Behavioral Ecology*, *19*(2), 448–455. https://doi.org/10.1093/beheco/arm144

Stone, D., Jepson, P., & Laskowski, R. (2002). Trends in detoxification enzymes and heavy metal accumulation in ground beetles (Coleoptera: Carabidae) inhabiting a gradient of pollution. *Comparative Biochemistry and Physiology - C Toxicology and Pharmacology*, *132*(1), 105–112. https://doi.org/10.1016/S1532-0456(02)00052-2

Tseng, M., Kaur, K. M., Soleimani Pari, S., Sarai, K., Chan, D., Yao, C. H., Porto, P., Toor, A., Toor, H. S., & Fograscher, K. (2018). Decreases in beetle body size linked to climate change and warming temperatures. *Journal of Animal Ecology*, *87*(3), 647–659. https://doi.org/10.1111/1365-2656.12789

Vucic-Pestic, O., Rall, B. C., Kalinkat, G., & Brose, U. (2010). Allometric functional response model: Body masses constrain interaction strengths. *Journal of Animal Ecology*, *79*(1), 249–256. https://doi.org/10.1111/j.1365-2656.2009.01622.x

Williams, G. (1959). Seasonal and Diurnal Activity of Carabidae, with Particular Reference to Nebria, Notiophilus and Feronia. *The Journal of Animal Ecology*, *28*(2), 309. https://doi.org/10.2307/2086

Wilman, H., J., B., J., S., C., de L. R., M., R., & W, J. (2014). EltonTraits 1.0 : Species-level foraging attributes of the world’s birds and mammals. *Ecology*, *95*(7), 2027.

Zygmunt, P. M. S., Maryański, M., & Laskowski, R. (2006). Body mass and caloric value of the ground beetle (Pterostichus oblongopunctatus) (Coleoptera, Carabidae) along a gradient of heavy metal pollution. *Environmental Toxicology and Chemistry*, *25*(10), 2709–2714. https://doi.org/10.1897/05-580R.1
